# The retrosplenial cortex and object recency memory in the rat

**DOI:** 10.1111/ejn.13577

**Published:** 2017-05-04

**Authors:** Anna L. Powell, Seralynne D. Vann, Cristian M. Olarte‐Sánchez, Lisa Kinnavane, Moira Davies, Eman Amin, John P. Aggleton, Andrew J. D. Nelson

**Affiliations:** ^1^ School of PsychologyCardiff UniversityTower BuildingPark PlaceCardiffCF10 3ATUK

**Keywords:** c‐*fos*, hippocampus, recognition memory, subiculum, temporal discrimination

## Abstract

It has been proposed that the retrosplenial cortex forms part of a ‘where/when’ information network. The present study focussed on the related issue of whether retrosplenial cortex also contributes to ‘what/when’ information, by examining object recency memory. In Experiment 1, rats with retrosplenial lesions were found to be impaired at distinguishing the temporal order of objects presented in a continuous series (‘Within‐Block’ condition). The same lesioned rats could, however, distinguish between objects that had been previously presented in one of two discrete blocks (‘Between‐Block’ condition). Experiment 2 used intact rats to map the expression of the immediate‐early gene c*‐fos* in retrosplenial cortex following performance of a between‐block, recency discrimination. Recency performance correlated positively with levels of c‐*fos* expression in both granular and dysgranular retrosplenial cortex (areas 29 and 30). Expression of c*‐fos* in the granular retrosplenial cortex also correlated with prelimbic cortex and ventral subiculum c*‐fos* activity, the latter also correlating with recency memory performance. The combined findings from both experiments reveal an involvement of the retrosplenial cortex in temporal order memory, which includes both between‐block and within‐block problems. The current findings also suggest that the rat retrosplenial cortex comprises one of a group of closely interlinked regions that enable recency memory, including the hippocampal formation, medial diencephalon and medial frontal cortex. In view of the well‐established importance of the retrosplenial cortex for spatial learning, the findings support the notion that, with its frontal and hippocampal connections, retrosplenial cortex has a key role for both what/when and where/when information.

## Introduction

Retrosplenial cortex is densely interconnected with the hippocampal formation and the anterior thalamic nuclei (van Groen & Wyss, 1990; van Groen & Wyss, 1992a,b; Van Groen & Wyss, 2003; Vann *et al*., 2009). The resulting interlinked network appears vital for rodent spatial memory (Sutherland & Hoesing, 1993). Lesions of retrosplenial cortex impair tests of spatial memory that involve allocentric cues, with examples coming from tasks in the water maze and radial‐arm maze, as well as impairing tests that rely on egocentric directional information and path integration (Sutherland *et al*., 1988; Warburton *et al*., 1998; Pothuizen *et al*., 2008; Keene & Bucci, 2009; Miller *et al*., 2014). Both temporary inactivation studies and electrophysiological recordings further implicate the retrosplenial cortex in spatial working memory and navigation (Cooper & Mizumori, 1999; Cho & Sharp, 2001; Cooper *et al*., 2001; Taube, 2007; Vedder *et al*., 2016). One proposal is that retrosplenial cortex forms part of a ‘where/when’ information network (Ritchey *et al*., 2015; Todd & Bucci, 2015).

In contrast, the importance of retrosplenial cortex for nonspatial memory remains unclear. One class of memory of particular interest is ‘what/when’ information as the combination of what/where/when is regarded as a hallmark of episodic‐like information (Clayton *et al*., 2007). It is already known that lesions in the rat hippocampus and anterior thalamic nuclei, along with prelimbic cortex, can disrupt the discrimination of nonspatial stimuli by their temporal order, i.e. ‘what/when’ information (Fortin *et al*., 2002; Hannesson *et al*., 2004a, b; Wolff *et al*., 2006; Barker *et al*., 2007; Barker & Warburton, 2011; DeVito & Eichenbaum, 2011; Albasser *et al*., 2012; Dumont & Aggleton, 2013). These findings prompted the need to know whether the rat retrosplenial cortex is also involved in this form of temporal memory. Initial evidence comes from the finding that rats with retrosplenial cortex lesions are impaired at discriminating time intervals (Todd *et al*., 2015) and from the description of a patient with left retrosplenial pathology who showed poor recency judgements (Bowers *et al*., 1988).

The present study used two complementary approaches. The first examined how retrosplenial cortex lesions affect behavioural tests of object recency (Experiment 1). The second mapped the expression of c*‐fos* in retrosplenial cortex following the performance of an object recency discrimination (Experiment 2). The immediate‐early gene c*‐fos* was chosen as it provides an indirect measure of neural activity (Herdegen, 1996; Chaudhuri, 1997; Tischmeyer & Grimm, 1999; Guzowski *et al*., 2005) and its expression is closely linked with spatial and contextual learning in both the hippocampus and retrosplenial cortex (Vann *et al*., 2000; Liu *et al*., 2012; Ramirez *et al*., 2013; Czajkowski *et al*., 2014).

In Experiment 1, rats received two classes of recency test. For the ‘Between‐Block’ recency test, the objects to be discriminated initially occurred within two separate, discrete blocks of sample trials. It is possible that such objects are distinguished by the temporal order of their respective blocks, making the subsequent recency judgements less reliant on detailed sequential information (DeVito & Eichenbaum, 2011; Templer & Hampton, 2013). For the ‘Within‐Block’ recency test, the rats selected between objects previously presented at different times within the same continuous series of sample trials (Shaw & Aggleton, 1993; DeVito & Eichenbaum, 2011; Dumont & Aggleton, 2013). While hippocampal lesions impair both Between‐Block and Within‐Block recency (Agster *et al*., 2002; Fortin *et al*., 2002; Albasser *et al*., 2010a,b, 2012; Barker & Warburton, 2011), anterior thalamic lesions appear more sensitive to tests of Within‐Block recency (Mitchell & Dalrymple‐Alford, 2005; Wolff *et al*., 2006; Dumont & Aggleton, 2013). Other studies with intact mice (DeVito & Eichenbaum, 2011) further support the notion that these two types of recency judgement may rely on different mnemonic processes.

For Experiment 2 (c*‐fos* mapping), tissue came from two groups of intact rats. One group experienced a Between‐Block condition (‘Recency Test’ group) whilst the other received a control procedure designed to match the sensorimotor demands of the Between‐Block recency test (Olarte‐Sánchez *et al*., 2014). This experiment examined whether retrosplenial c*‐fos* expression correlates with recency memory performance and how this expression relates to other brain sites involved in recency memory, namely the anterior thalamic nuclei, hippocampus and prelimbic cortex (Hannesson *et al*., 2004b; Mitchell & Dalrymple‐Alford, 2005; Barker *et al*., 2007; Albasser *et al*., 2012; Dumont & Aggleton, 2013).

Given the dense interconnectivity between the retrosplenial cortex, hippocampus and anterior thalamic nuclei, alongside the involvement of the latter two regions in recency memory, we anticipated that temporal order discriminations would be affected by retrosplenial cortex lesions (Experiment 1). It was further predicted that such recency judgements would engage the retrosplenial cortex, as measured by c‐*fos* expression, with related changes in interconnected sites.

## Materials and methods

### Experiment 1: Recency judgements in rats with retrosplenial cortex lesions

#### Subjects

Experiment 1 involved a single cohort of 30 male Lister Hooded rats (ENVIGO, Bicester, UK). At the time of surgery, the rats weighed 309–356 g. Animals were housed in groups of four under diurnal light conditions (14 h light/10 h dark). All behavioural testing was carried out during the light phase. Prior to surgery, the rats were handled daily for a week and then randomly assigned to one of two surgical groups: retrosplenial cortex lesions (RSC, *n* = 15) or surgical sham (Sham, *n* = 15). All procedures in Experiment 1 (and Experiment 2) were conducted in accordance with the UK Animals (Scientific Procedures) Act, 1986 and EU directive (2010/63/EU), as well as receiving approval from local ethical committees at Cardiff University.

#### Surgical procedures

Prior to the induction of anaesthesia, animals received an intraperitoneal injection (i.p) of atropine (0.06 mL of a 600 μg/mL solution, Martindale Pharma, Brentwood, UK) and subcutaneous Metacam (0.03 mL of a 5 mg/mL solution, Buehringer Ingelheim Lid, Bracknell, UK). Rats were deeply anaesthetised (1 mL/kg, i.p. injection) with 6% sodium pentobarbital solution (Ceva Animal Health, Libourne, France). Anaesthesia was then maintained with isoflurane (~0.5%) in O_2_ for the duration of the surgery. Rats were placed in a stereotaxic frame (David Kopf Instruments, Tujunga, CA, USA) with the nose‐bar set at +5.0. Lidocaine (Xylocaine, AstraZeneca, Luton, UK) was administered by subcutaneous injection to the scalp, which was then incised and retracted. A bilateral craniotomy, extending from bregma to lambda, exposed the cortex along the midline. Lesions were produced by injecting a solution of 0.09 m *N*‐methyl‐d‐aspartic acid (NDMA; Sigma‐Aldrich, Gillingham, UK) dissolved in phosphate buffer (pH 7.2), via a 1‐μL Hamilton syringe (Bonaduz, Switzerland), at six bilateral sites within retrosplenial cortex. After each injection, the needle was left *in situ* for 5 min.

Anterior‐posterior (AP) coordinates were measured (in mm) from bregma, the medio‐lateral (ML) coordinates (in mm) from the sagittal sinus, and the dorso‐ventral (DV) coordinates (in mm) with reference to the level of the dura. The stereotaxic coordinates at each of the six sites were as follows: (#1) −1.8 (AP), ± 0.5 (ML), −1.0 (DV); (#2) −2.8 (AP), ± 0.5 (ML), −1.1 (DV); (#3) −4.0 (AP), ± 0.5 (ML), −1 (DV); (#4) −5.3 (AP), ± 0.5 (ML), −2.5 (DV); (#5) −5.3 (AP), ± 0.9 (ML), −1.4 (DV); (#6) −6.6 (AP), ± 0.9 (ML), −1.8 (DV). A volume of 0.25 μL of NDMA was injected at sites #1–3 and 0.27 μL at sites #4–6.

On completion of the surgery, the skin was sutured and Clindamycin antibiotic powder (Pfizer, Walton Oaks, UK) was applied topically and animals were rehydrated with glucose saline (5 mL, s.c.). Surgical shams received the identical procedure except that the needle was not lowered into the cortex and no NDMA injections were made. All rats received a minimum of 10 days postoperative recovery.

#### Apparatus

##### Bow‐tie maze

**Figure 1:**
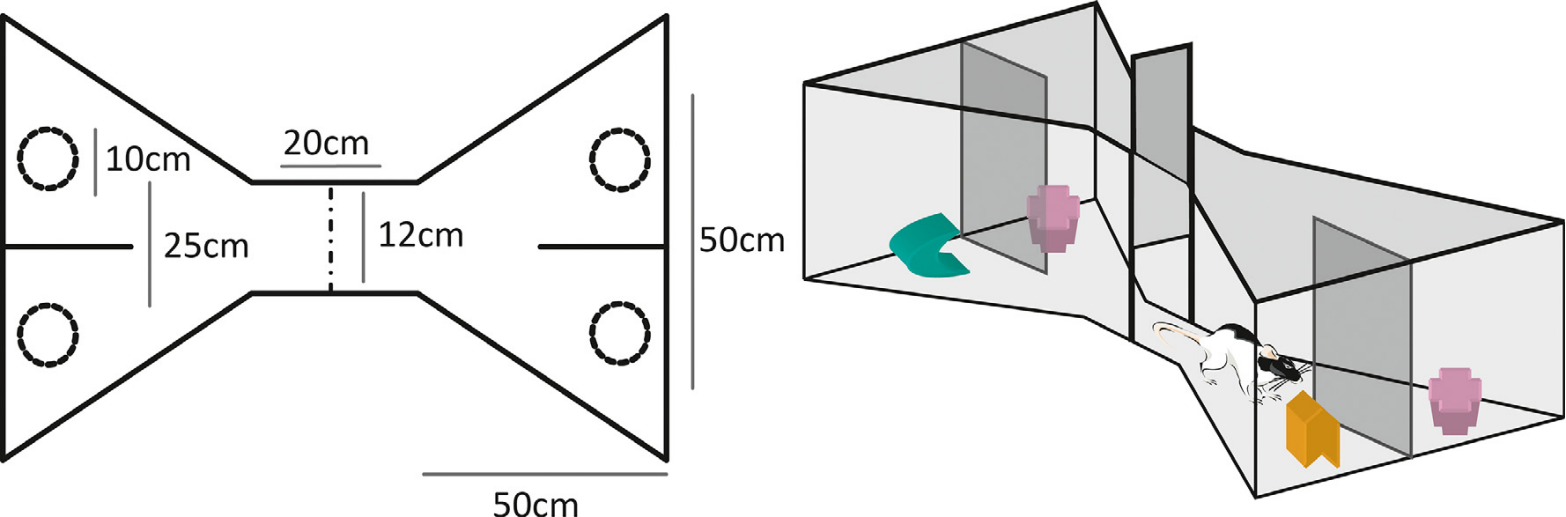
The shape and dimensions (cm) of the bow‐tie maze used in Experiments 1 and 2. The circles in the plan (left) depict the locations of the food wells, which is where objects were placed. [Colour figure can be viewed at wileyonlinelibrary.com].

All behavioural testing was conducted in an aluminium bow‐tie shaped maze (120 cm long, 50 cm wide and 50 cm high; Fig. 1). The triangular chambers at each end of the maze were joined together at their apices by a linking corridor (12 cm wide). In the centre of the corridor was an opaque sliding door that could be raised by the experimenter, enabling the rat to move from one end of the maze to the other. At the far wall of each of the triangular chambers, there were two food wells (3.5 cm in diameter and 2 cm deep), separated by a short, opaque wall that protruded 15 cm from the middle of the end wall. The two food wells were 25 cm apart. Objects covered these two food wells during the experiment. All sessions were video‐recorded using a single, ceiling mounted camera.

##### Objects

**Figure 2:**
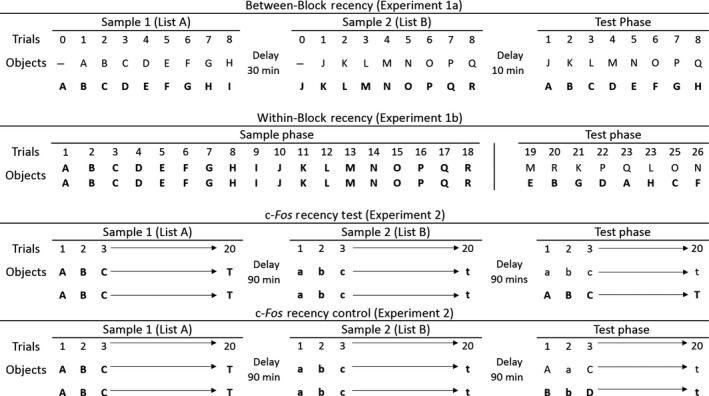
Schematic diagram showing the sequence of object presentation in Experiments 1a, 1b and 2. Different objects are represented by different letters and by changes in case (upper or lower). To represent the first presentation of an object in the Sample Phase (i.e. when novel), the letter is in bold. Bold typeface in the Test Phase reflects that object in the trial that was first encountered longer ago in time. The Table shows the order of object presentation across the different Phases of the Between‐Block (Experiment 1a) and Within‐Block (Experiment 1b) recency procedures. The Within‐Block condition (Experiment 1b) did not involve a separate Sample Phase prior to the Test Phase as these phases were integrated into a single, continuous session. In the Test Phases in all four conditions (Experiments 1, 2) each trial involved two different, familiar objects from different times in the past.

For Experiment 1a (Between‐Block recency), each of the two sessions required 18 pairs of identical objects (see Fig. 2). These objects varied in size, shape, colour and texture, but lacked an obvious odour. New objects were used for each session. The presentation order of these object pairs was counterbalanced across animals. The objects used were cleaned thoroughly with alcohol wipes between the Sample and Test Phases to avoid scent marking.

For Experiment 1b (Within‐Block recency), a different set of 18 objects was used in each of the two sessions (Fig. 2). The presentation order of these objects was counterbalanced, so that half of the rats were presented with the objects in one order (e.g. A–R) and the other half experienced the list in the reverse order (e.g. R–A). New identical objects were used in the Test Phase to avoid scent marking.

All objects were large enough to cover one food well but light enough for the rats to displace with ease. For both experiments, the relative left and right positioning of old and recent items was counterbalanced.

#### Behavioural procedures

##### Habituation and pre‐training

Testing started approximately 4 months after surgery. Prior to these experiments, all animals had completed a single appetitive operant learning task. Animals were habituated to the bow‐tie maze for 7 days. By the end of pre‐training, all rats would run from one end of the maze to the other and displace objects covering the food wells to obtain a sucrose pellet reward (45 mg; Noyes Purified Rodent Diet, UK). On the first pre‐training day, rats were placed in the maze in pairs for 20 min and allowed to explore and consume sucrose pellets scattered across the floor and in the food wells. On the second day, rats were placed in the maze individually for 10 min to run back and forth between the two chambers and collect rewards located in the food wells. From the third day, the sliding door was introduced in order to control movement from one compartment to the other. On subsequent days, four identical plastic blocks were introduced to the two chambers and then gradually moved over the food wells. By the end of the 10 min session, the rats had learnt to push aside the blocks to obtain the food reward. On subsequent sessions, three other pairs of objects were introduced that varied in size, shape, colour and weight. These three objects were only used during pre‐training and not during the experiment proper.

#### Experiment 1a: Between‐Block recency

Each recency test consisted of a preference choice between two objects, one from each of two lists that were separated by a period back in the home cage. For this reason, each test session consisted of two Sample Phases and one Test Phase (see Fig. 2). Each Sample Phase comprised eight trials. At the start of each Sample Phase, rats were placed at one end of the maze (the same end for all rats), which was empty. On every subsequent trial, each food well contained a single sucrose pellet, which was covered by an object. The Test Phase was identical to the Sample Phases except that all objects used in the Test Phase were from the preceding Sample Phases. The rats completed two test sessions, which were between five and 12 days apart.

To start Sample Phase 1, the sliding door was raised, enabling access to the other end of the maze, which contained a novel object (A) covering one food well and a plastic block (used in pre‐training) covering the other (Trial 0). The rats were given 1 min to explore the objects and retrieve the food pellets that they covered, before the door was raised again to allow access back to the first compartment. For Trial 1, this compartment now contained a duplicate copy of object A (familiar) alongside object B (novel). After 1 min, the door was again raised. On Trial 2, the rat was allowed to explore a duplicate of object B (familiar) and object C (novel). Rats completed eight such sample trials (in addition to the initial trial 0) before being returned to the carrying box for 30 min (see Fig. 2).

Sample Phase 2 started after this 30 min interval and was identical in structure to Sample Phase 1, except that new objects were used (objects J‐R). After eight trials, the rats were again returned to the carrying box, this time for 10 min, after which the Test Phase commenced. In this way, the rats sampled two distinct blocks of objects (Fig. 2).

The Test Phase followed a similar format to the Sample Phases, except that now the pairs of objects presented in each trial consisted of one object from Sample Phase 1 (objects A‐H; *old*) and one object from Sample Phase 2 (objects J‐Q; *recent*). For example, in Trial 1 object A (*old*) and object J (*recent*) were presented together for 1 min.

It should be noted that both Sample Phase 1 and 2 were designed to also provide a measure of recognition memory (novel vs. familiar). Every Sample Phase trial consisted of one novel object and one familiar object (from the previous trial). By recording the exploration times for each object during the sample trials, it was possible to determine if rats preferentially explored the novel object within each trial pairing (see Fig. 2, Experiment 1a).

#### Experiment 1b: Within‐Block recency

Each recency test consisted of a preference choice between two previously explored objects, drawn from a continuous sequence of sample presentations. For this reason, each session consisted of an 18‐trial Sample Phase and an eight‐trial Test Phase within a continuous block of trials (Fig. 2). Unlike Experiment 1a, the rat remained in the bow‐tie maze throughout.

In common with Experiment 1a, each session began by placing the rat into an empty compartment of the bow‐tie maze. The sliding door was then raised to allow access to the other compartment, which contained two identical objects (A1, A2), each covering a food well (Sample Trial 1). After a minute had elapsed, the sliding door was opened and the rat returned to the first compartment, which now contained two copies of object B (B1, B2). After 18 Sample Phase trials, the Test Phase began immediately. The rats received two sessions, with no object repeats across the sessions. The sessions were between 15 and 22 days apart.

On each Test Phase trial, the rat was allowed to explore two objects of different recency, each covering a food reward. For example, copies of Object E (*old*) and Object M (*recent*) would be presented together (Fig. 2). After a minute, the linking door was raised and the rat entered the other compartment, allowing it to explore a different *old*/*recent* object pairing. The number of interleaving Sample Phase trials between the two objects in the Test Phase was set at 3, 7, 11 or 15, arranged in a balanced order. Every item was experienced in the same compartment end of the maze for both the Sample and Test Phases.

#### Behavioural analysis

The times spent exploring each object was scored from the video recordings. Exploration was defined as time when the animal directed its nose towards the objects at a distance of < 1 cm or when it touched the object with its nose or the paws (including pushing). Time spent chewing, sitting on or turning around the item was not classified as exploration. The videos were scored blind to lesion group assignment.

In standard tests of object recognition, the greater time spent exploring a novel object when compared to a familiar object is used as a proxy measure of successful recognition memory. Here, when testing recency memory, the contrast is made between the exploration times for the older object (i.e. treated as if it were the ‘novel’ object) with those of a more recently presented object (i.e. treated as if it were the ‘familiar’ object). This contrast follows numerous experiments that have confirmed how rats prefer to explore the least familiar object of a pair (Hannesson *et al*., 2004b; Mitchell & Dalrymple‐Alford, 2005; DeVito & Eichenbaum, 2011; Albasser *et al*., 2012). Thus, for the Test Phase, index D1 is the time spent exploring the more recent (‘familiar’) object subtracted from the time spent exploring the older (‘novel’) object. This difference was summed across trials to give a cumulative D1 value. The D2 index was then calculated by dividing this cumulative D1 score by the total time spent exploring all objects across the multiple trials. Consequently, a positive score (maximum +1) indicates a preference for the older item, while a score of 0 reflects no preference. Furthermore, the Sample Phases in Experiment 1a were designed in such a way as to provide an index of novel object recognition memory. Therefore, the D1 and D2 indices were also calculated for these trials as for standard tests of object recognition.

For both Experiments 1a and 1b, data were averaged (mean) across the two sessions. Differences between the experimental groups in their final D2 scores and total exploration times (i.e. the total amount of time spent exploring both objects in each pair) were assessed using *t*‐tests. Above chance discrimination was defined as a final D2 score significantly > 0, as assessed using a one‐sample *t*‐test (two‐tailed).

#### Histology

Following behavioural testing, the animals were given a lethal overdose of Euthatal (200 mg/mL sodium pentobarbital, Marial Animal Health, Harlow, Essex, UK) via intraperitoneal injection and intracardially perfused with 0.1 m phosphate buffered saline (PBS), followed by 4% paraformaldehyde in 0.1 m PBS (PFA). The brains were extracted from the skull and placed on a shaker to postfix in PFA for 4 h, after which they were placed in 25% sucrose overnight. The brains were frozen on a microtome (Leica, UK) and sectioned at 40 μm in the coronal plane. A one‐in‐four series of sections was mounted onto gelatin‐coated slides and stained with cresyl violet, a Nissl stain, for histological assessment of lesion placement and size.

### Experiment 2: *c‐fos* expression in the retrosplenial cortex and associated areas after performing a recency memory task

This experiment took advantage of a previous study of recency memory (Olarte‐Sánchez *et al*., 2014) but analysed new regions (the retrosplenial cortex) and considered novel aspects of the behavioural performance. An integral feature of this experiment was the need to separate the Sample Phase from the Test Phase. This separation is required so that the c*‐fos* activity only reflects the Test Phase, i.e. the recency memory test. The expression of Fos, the protein product of the immediate‐early gene c*‐fos*, typically peaks between 60 and 120 min after exposure to the inducing stimulus (Bisler *et al*., 2002; Zangenehpour & Chaudhuri, 2002). For this reason, a Between‐Block design was used with an interval of 90 min between the second Sample Phase and the Test Phase, followed by a further 90 min delay before perfusion.

#### Subjects

A total of 18 naïve, male, Lister Hooded rats (Harlan, Bicester, United Kingdom) were housed in pairs under diurnal conditions (12‐h light/dark). At the start of testing, rats were approximately 10 weeks old and weighed between 277 and 355 g. During behavioural testing, the rats were food restricted and maintained at ~85% of their free‐feeding body weight. Water was available *ad libitum*. Pairs of rats housed together were randomly divided into two groups (both *n* = 9): Recency Test and Recency Control.

#### Apparatus

The apparatus (a bow‐tie maze) was the same as that used in Experiment 1. Experiment 2 used 40 different object pairs for the sample trials (see Fig. 2). Each pair was identical but the pairs differed from one another in their colour, shape, size and texture. The test trials involved duplicates of these same objects. The objects were cleaned with alcohol wipes after each session.

#### Behavioural procedures

Rats were first habituated to the bow‐tie maze (see Experiment 1). Both the Recency Test and the Recency Control consisted of two Sample Phases and one Test Phase. Each of these Phases was separated by a 90‐min interval. Each Phase consisted of twenty 1‐min trials. The sample trials followed essentially the same structure as Experiment 1a, except that each sample trial consisted of two identical objects and a total of 20 pairs were used (Fig. 2). All rats completed one session.

##### Recency test

During the Test Phase trials, rats were presented with two different, familiar objects: one duplicate object from the first Sample Phase (old) and one duplicate from the second Sample Phase (recent) (Fig. 2). These object pairs were presented in the same order as in their respective Sample Phases, such that Test Trial 1 involved the item from Trial 1 of Sample Phase 1 (object A) and the item from Trial 1 of Sample Phase 2 (object a). This arrangement ensured that the objects from the two Sample Phases were always separated by the same interval (110 min). Placement of the old and recent objects on the left or right was counterbalanced.

##### Recency control

The rats in the Recency Control group received two Sample Phases, identical to those completed by the Recency Test group. Again, in the Test Phase, the rats were presented with pairs of familiar, different objects. However, the object pairs were now taken from successive trials in the same Phase (Fig. 2). For example, in Trial 1 of the Test Phase, the object from Trial 1 (object A) and from Trial 2 (object B) of the first Sample Phase were presented together. In Trial 2, the objects presented together were from Trial 1 (object a) and Trial 2 (object b) of the second Sample Phase (Fig. 2). This design reduced the recency differences between test objects to a few seconds, whilst keeping all other conditions as for the Recency Test.

##### Immunohistochemistry

After behavioural testing, rats were placed in a dark holding room for 90 min before being perfused. This 90‐min interval matched the expected expression peak of Fos (Bisler *et al*., 2002; Zangenehpour & Chaudhuri, 2002). The rat brains were extracted and sectioned, as described in Experiment 1.

A 1‐in‐4 series of 40 μm coronal sections was processed concurrently in experimental pairs (i.e. one Recency Test rat and one Recency Control rat). Sections were first washed six times in 0.2% Triton‐X 100 in 0.1 m PBS (PBST), once in 0.3% H_2_O_2_ in PBST and four times in PBST (these and all washes were 10 min). Sections were then incubated in primary antibody solution, rabbit‐anti‐*c‐*Fos diluted in PBST (1:3000; Ab‐4, Calbiochem), for 48 h at 4 °C. After being washed four more times in PBST, the sections were incubated in secondary antibody solution, biotinylated goat‐anti‐rabbit (1:200; Vector Laboratories) diluted in 1.5% normal goat serum in PBST for 2 h at room temperature. Following another four PBST washes, the sections were incubated in avidin‐biotinylated horseradish peroxidase complex in PBST (Elite kit, Vector Laboratories) for 1 h at room temperature. Finally, sections were washed four times in PBST, then twice in 0.05 m Tris buffer, before a diaminobenzidine (DAB Substrate kit; Vector Laboratories) was used to chromogenetically visualise the reaction. The reaction was stopped using cold PBS. Sections were mounted onto gelatin‐coated slides, dehydrated and coverslipped.

##### Fos‐positive cell counts

Digital data from the regions of interest (ROIs) were captured across both hemispheres from four consecutive sections (each 120 m apart) using a Leica DMRB microscope and an Olympus DP70 Camera. Cells immunopositive for the protein product of c*‐fos* (Fos) were counted using ANALYSIS^D software (Soft‐Imaging Systems; Olympus, Southend, UK). This software selects and counts cells automatically, avoiding experimenter bias. In addition, the experimenter was blind to the group conditions. While stereological methods are essential in order to derive accurate absolute cell counts (Coggeshall & Lekan, 1996), the goal of the present study was to compare relative Fos‐positive cell counts between areas and between the two conditions. For this purpose, automated cell counting is appropriate when certain conditions are met. These conditions include no systematic changes in the volume or packing of the neurons across the two groups along with random tissue sampling (Coggeshall & Lekan, 1996; Mura *et al*., 2004). These conditions were present in the current study.

Counts of labelled cells in each region of interest were determined by counting those nuclei (mean feret, a measure of particle size, of 4–20 μm) stained above a threshold of greyscale intensity that was above background levels. Cell counts in the retrosplenial cortex were made with a frame area of 0.84 × 0.63 mm, which enabled all laminae to be included in one image.

#### Regions of interest

**Figure 3:**
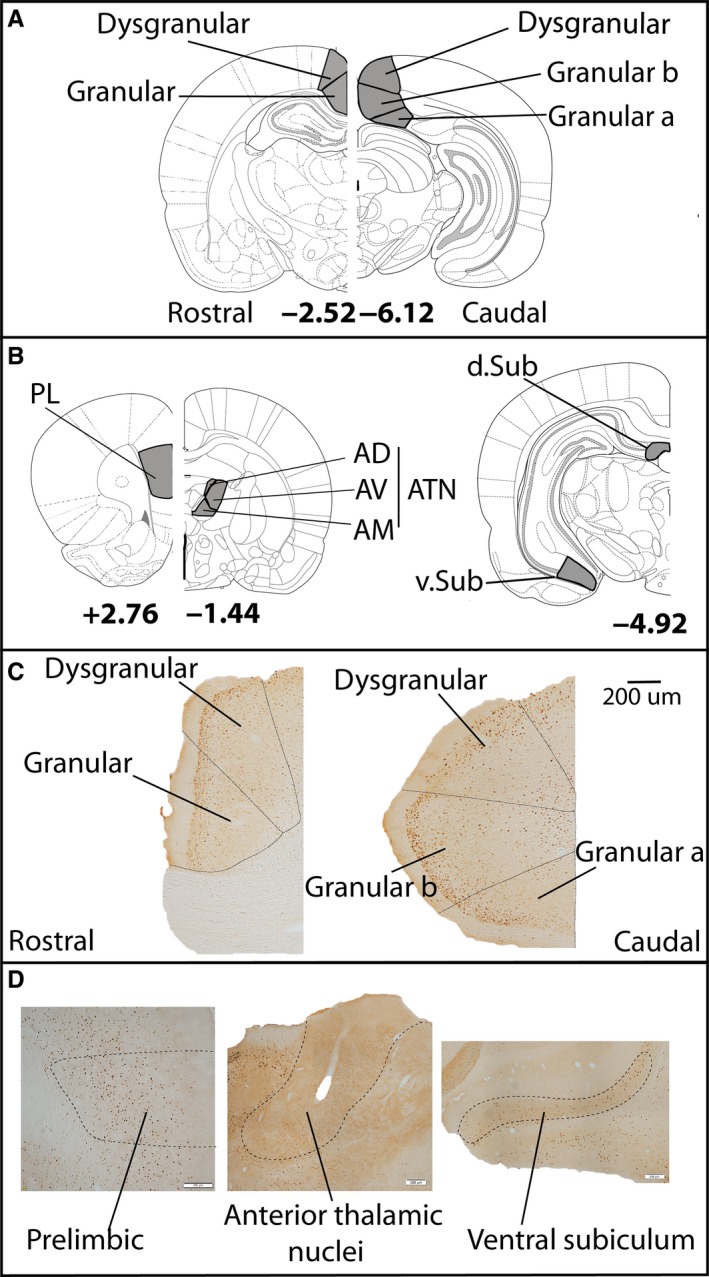
The regions of interest within the retrosplenial cortex (A) and related areas (B). Examples of sections stained for Fos protein in retrosplenial cortex (C) and related areas (D). Abbreviations: ATN, anterior thalamic nuclei, consisting of the AD (anterodorsal), AM (anteromedial) and (AV) anteroventral nuclei; PL, prelimbic cortex; Sub, subiculum (d, dorsal, v, ventral). The other labels refer to different subregions within retrosplenial cortex (Van Groen & Wyss, 2003). The numbers refer to distance from bregma (Paxinos & Watson, 2005). Scale bar = 200 μm. [Colour figure can be viewed at wileyonlinelibrary.com].

The various retrosplenial regions of interest (ROIs) are illustrated in Fig. 3A. Separate cell counts were taken from five retrosplenial areas. In each area, the counts for layers II‐III (superficial) and V‐VI (deep) were calculated separately. The nomenclature for these granular and dysgranular areas follows that of van Groen & Wyss (1990). For sections caudal to the splenium, a distinction was made between granular cortex a and granular cortex b.

In addition, Fos‐positive cell counts were analysed from three areas closely related to retrosplenial cortex (Fig. 3B). These additional analyses were based on prior Fos‐positive cell counts (Olarte‐Sánchez *et al*., 2014). The hippocampal formation counts were from the dorsal subiculum and ventral subiculum. This hippocampal region was selected as it is closely connected not only with the retrosplenial cortex, but also with the anterior thalamic nuclei and prelimbic cortex (Dillingham *et al*., 2015a,b; Christiansen *et al*., 2016). Further cell counts were from the prelimbic cortex, the anterodorsal (AD), anteromedial (AM) and anteroventral (AV) thalamic nuclei. The counts from the various anterior thalamic nuclei (ATN) were combined in order to reduce the numbers of comparisons.

### Statistical analysis

#### Behavioural analysis

Final D2 scores and total exploration times were calculated and compared as for Experiment 1.

#### IEG analyses

Initial analyses compared the raw counts of Fos‐positive cells in the regions of interest.

In order to protect against Type I errors, the number of comparisons was reduced. For this reason, regions of interest that involved the same retrosplenial subregion were grouped and then analysed with a repeated measures anova. Specifically, Fos cell counts for those sections rostral and caudal to the splenium were combined, as were counts from granular cortex a and b. The divisions between deep and superficial layers, and between granular and dysgranular cortex, were preserved. Thus, the final groupings for analysis were: Granular deep, Granular superficial, Dysgranular deep and Dysgranular superficial.

A Spearman Rank correlation coefficient assessed the relationship between the Fos cell counts for each of the four subarea groupings and the final D2 index. This analysis was conducted separately for the Recency Test and Control groups. Bonferroni corrections reduced the Type I error rate.

## Results

### Experiment 1: Recency judgements in rats with retrosplenial cortex lesions

#### Histology

**Figure 4:**
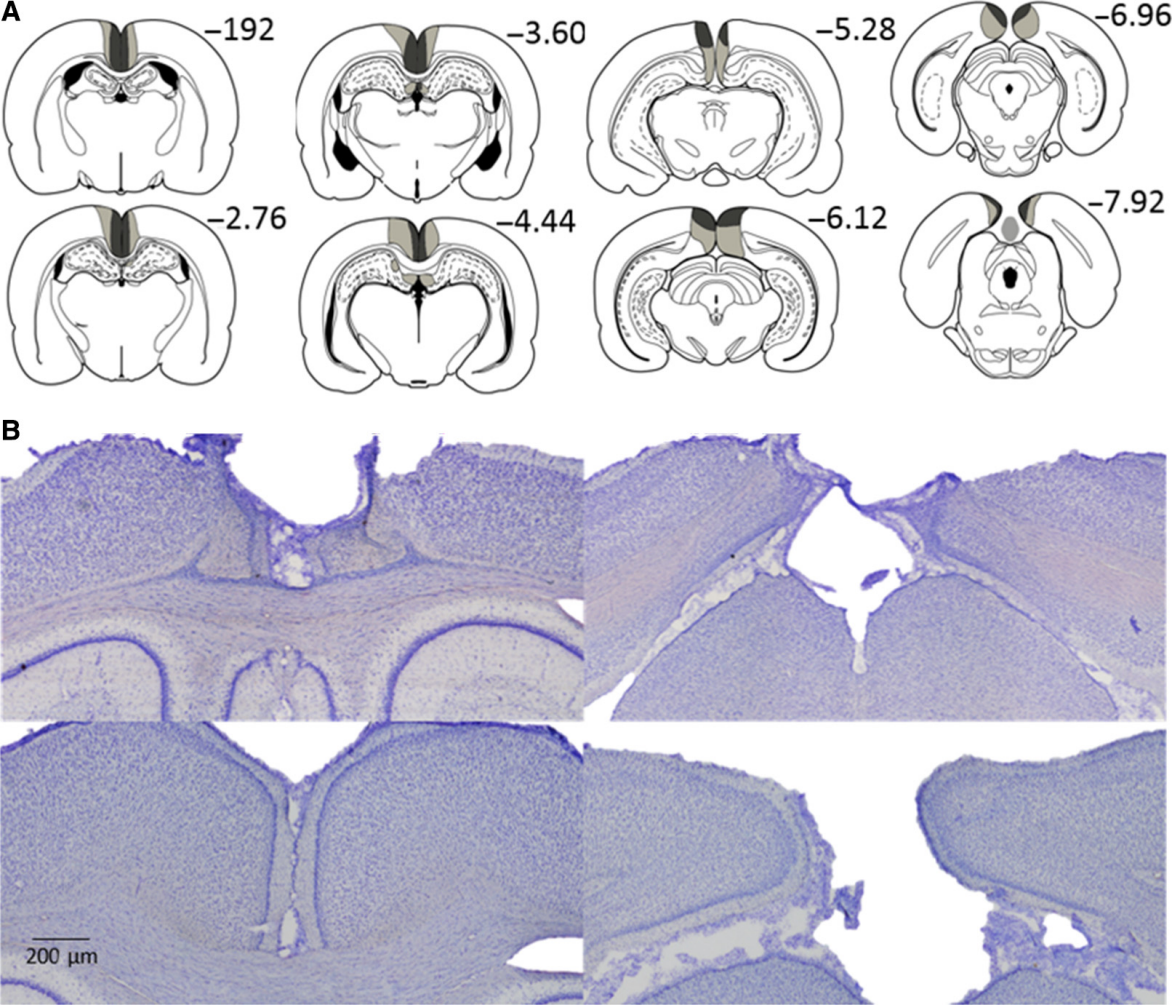
Location and extent of the retrosplenial cortex lesions (Experiments 1 a and b). A. The largest (pale grey) and smallest (dark grey) cortical lesions on a series of coronal sections. B. Photomicrographs from a representative lesion (top row) and a surgical sham case (bottom row). (See Fig. 3 for the divisions within retrosplenial cortex). Scale bars represent 200 μm. The numbers refer to the approximate distance in mm of each section caudal to bregma (Paxinos & Watson, 2005). [Colour figure can be viewed at wileyonlinelibrary.com].

Two animals from the RSC group were excluded from analysis due to bilateral damage in dorsal CA1. Of the remaining 13 animals, five had lesions that were centred on the dysgranular cortex. One of these five cases had almost complete sparing of the granular cortex both anterior and posterior to the splenium. In the other four of these cases, some cell loss was also evident in the deeper layers of the granular cortex. A sixth animal had appreciable unilateral sparing in granular retrosplenial cortex anterior to the splenium (Fig. 4).

The remaining seven RSC animals showed considerable bilateral cell loss in both the granular and dysgranular cortices, both anterior and posterior to the splenium (Fig. 4). In some cases, this cell loss was particularly evident in the more superficial cell layers (Fig. 4). In two of these cases, there was a small amount of sparing in granular retrosplenial cortex close to the anterior cingulate border. Caudal to the splenium, there was often some sparing in the most caudal parts of area Rga. Three cases had minimal unilateral cell loss limited to the dorsal medial CA1 below the rostral retrosplenial cortex. In one case, the lesion encroached into caudal anterior cingulate cortex. In total, the data from 13 RSC and 15 Sham animals were compared.

#### Experiment 1a: Between‐Block recency

**Figure 5:**
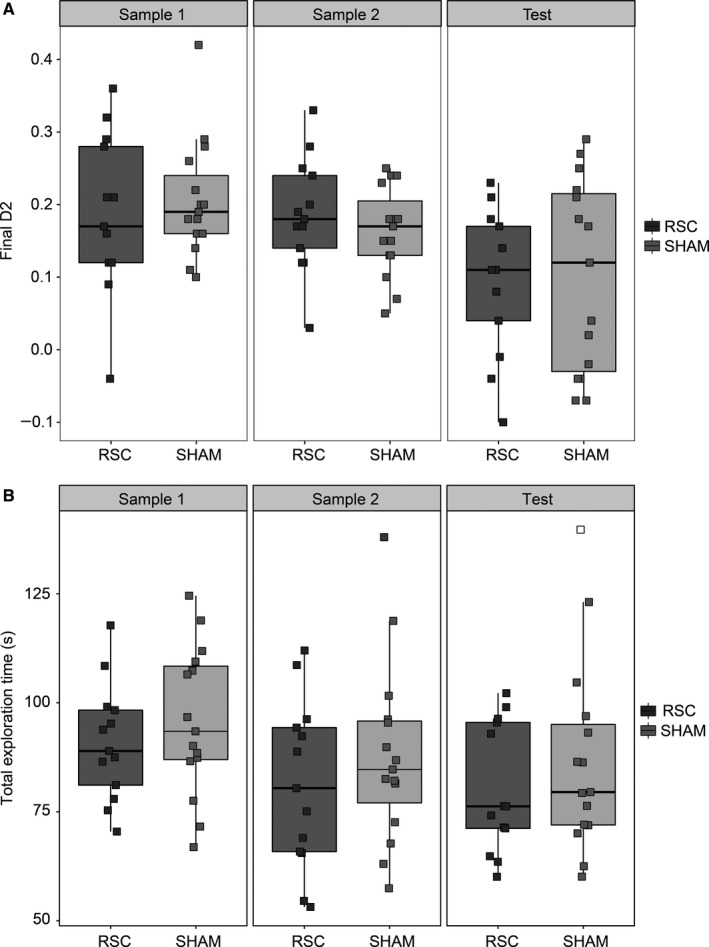
Experiment 1a, Between‐Block Recency. (A) The final D2 scores from Sample Phase 1 and 2 (recognition), as well as the Recency Test Phase in both groups. (B) Total exploration time during the two Sample Phases and the Test Phase in the retrosplenial cortex lesion (RSC) and Sham groups. The central line on each box shows the median value. The box extends from the first to the third quartile. The upper and lower whiskers extend 1.5× interquartile range. Note that data points were ‘jittered’ on the *x*‐axis, by adding random noise to the values of the categorical variable, in order to better visualise overlapping data points.

Rats were tested for two sessions and the data from these sessions were combined prior to statistical analysis. There was no difference in total exploration time between the two groups during either Sample Phase 1, Sample Phase 2 or the Test Phase (all *t *<* *1; Fig. 5B). Nor did the two groups differ in the degree to which they discriminated between old and recent items, as indexed by the D2 ratio from the Test Phase (*t *<* *1; Fig. 5A). Both Sham (*t*_14_ = 3.02, *P *=* *0.009) and RSC (*t*_12_ = 3.47, *P *=* *0.005) animals showed above chance discrimination at test (i.e. D2 > 0).

The two groups did not differ in their discrimination between novel and familiar items during either Sample Phase 1 or Sample Phase 2, as measured by the D2 ratio (both *t *<* *1; Fig. 5A) and both groups showed above chance recognition performance during each sample phase (smallest *t* value [RSC Sample 1]: *t*_12_ = 6.06, *P *<* *0.001; Fig. 5A).

#### Experiment 1b: Within‐Block recency

The test trials in Experiment 1b differed in terms of the number of interleaving items presented between the two objects during the Sample Phase (i.e. 3, 7, 11, 15, Fig. 2). There was, however, no main effect of trial type (i.e. the number of interleaving items) on D2 scores (*F *<* *1) and no interaction between trial type and surgical group (*F*_3,26_ = 1.35, *P *=* *0.27). Therefore, the data were collapsed across trial type for subsequent analysis.

**Figure 6:**
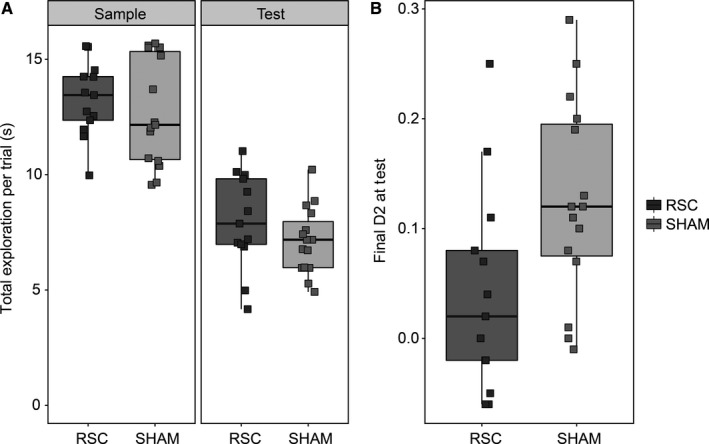
Experiment 1b, Within‐Block Recency. (A) Total exploration time per trial during the Sample and Test Phases in the retrosplenial (RSC) lesion and Sham groups. (B) The final D2 scores from the Test Phase in both groups (B). The central line on each box shows the median value. The box extends from the first to the third quartile. The upper and lower whiskers extend 1.5× interquartile range. Note that data points were ‘jittered’ on the *x*‐axis (see Fig. 5).

There was no difference in total exploration times between the two groups during either the Sample Phase (*t *<* *1) or Test Phase (*t*_26_ = 1.28, *P *=* *0.21). However, there was a significant group difference in the final D2 scores for the recency discriminations (*t*_26_ = 2.45, *P *=* *0.021, two‐tailed, Fig. 6B). Consistent with this group difference, the Sham group successfully discriminated old from recent objects (*t*_14_ = 5.39, *P *<* *0.001), while the RSC group failed to discriminate the objects based on their relative recency (*t*_12_ = 1.58, *P *=* *0.14).

As it was not possible to assess recognition discrimination during the Sample phase in this experiment (because identical objects were presented on each trial), we looked at the difference in exploration time per trial during the Sample (novel) and Test (familiar) Phases, as a proxy measure of object recognition. Both groups spent more time exploring the objects when they were encountered for the first time (*F*_1,26_ = 299.5, *P *<* *0.001, Fig. 6A), suggesting the rats discriminated the novel from the familiar objects. Importantly, there was no group difference (*F*_1,26_ = 1.20, *P *=* *0.28) and no interaction (*F *<* *1) with respect to the Sham and RSC animals.

As the D2 scores of the Sham animals were comparable between Experiments 1a and 1b, the respective discrimination levels of the two groups were compared across the two experiments in an anova. There was no main effect of experiment (*F *<* *1), no interaction between group and experiment (*F*_1,26_ = 1.79, *P *=* *0.19) and no overall effect of group (*F*_1,26_ = 2.83, *P *=* *0.11).

### Experiment 2: *c‐fos* expression in the retrosplenial cortex and associated areas after performing a recency memory task

#### Behaviour

**Figure 7:**
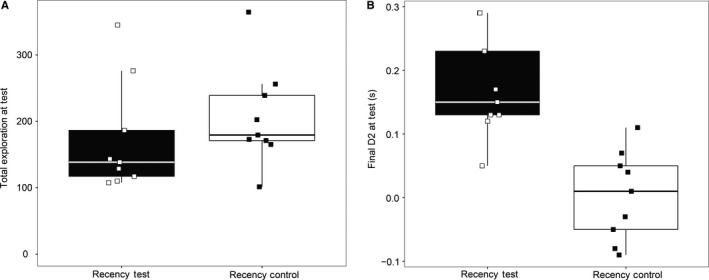
Experiment 2, c‐fos expression associated with recency memory. (A) Total times spent exploring objects during the Test Phase of the Recency Test and the Recency Control conditions. (B) Final D2 scores for the Recency Test and the Recency Control. The central line on each box shows the median value. The box extends from the first to the third quartile. The upper and lower whiskers extend 1.5× interquartile range. Note that data points were ‘jittered’ on the *x*‐axis (see Fig. 5).

The main behavioural findings have been published previously (Olarte‐Sánchez *et al*., 2014). There was no group difference in the total exploration times of the Recency Test and Control groups during the Test Phase (*t *<* *1; Fig. 7A). However, as expected, the Recency group were significantly better at discriminating between old and recent objects than the Control group, as shown by comparing the final D2 scores (*t*_16_ = 4.77, *P *<* *0.001; Fig. 7B). The Recency Test group showed above chance temporal discriminations (one‐sample *t*_8_ = 6.42, *P* < 0.001). In contrast, rats in the Recency Control group were unable to discriminate objects based on their relative recency (*t*_8_ = 0.17, *P *=* *0.87).

Additional analyses showed that the Recency Control group had similar total exploration times for those trials in the Test Phase that involved objects from Sample Phase 1, compared with those trials using objects from Sample Phase 2 (*t*_8_ = 1.51, *P *=* *0.17). This lack of difference, along with the finding that the D2 recency scores did not differ for these two sets of trials (*t *<* *1), provides further evidence that the rats in this control condition did not perform recency judgements.

#### *c‐fos* activation

##### Group differences

**Figure 8:**
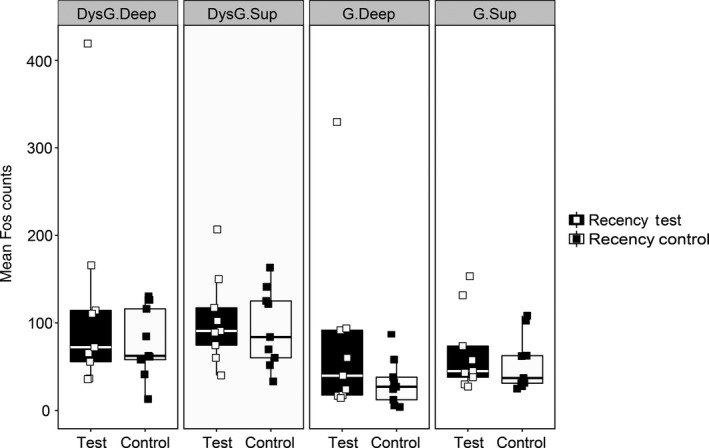
Experiment 2, c‐fos expression associated with recency memory. Comparison of the Fos‐positive cell counts in each of the major retrosplenial subareas for the Recency Test and Recency Control groups. Error bars show ± SEM. Abbreviations: DysG.Deep: Dysgranular cortex, deep layers; DysG.Sup: Dysgranular cortex, superficial layers; G.Deep, Granular cortex, deep layers; G.Sup: Granular cortex, superficial layers. The central line on each box shows the median value. The box extends from the first to the third quartile. The upper and lower whiskers extend 1.5× interquartile range. Note that data points were ‘jittered’ on the *x*‐axis (see Fig. 5).

Figure 3C and D illustrate the appearance of Fos‐positive cells in the regions of interest. Although Fos cell counts were higher in sections from caudal retrosplenial cortex than in rostral sections (*F*_*1,16*_ = 8.82, *P *=* *0.009), this effect did not interact with test condition (*F*_*1,16*_ = 1.41, *P *=* *0.25). Therefore, all subsequent analyses used the subarea groupings described above (i.e. Granular deep, Granular superficial, Dysgranular deep and Dysgranular superficial). There was no overall effect of test condition on Fos counts (*F *<* *1) and no interaction between region and group (*F *<* *1; Fig. 8).

##### Correlations with behaviour

**Figure 9:**
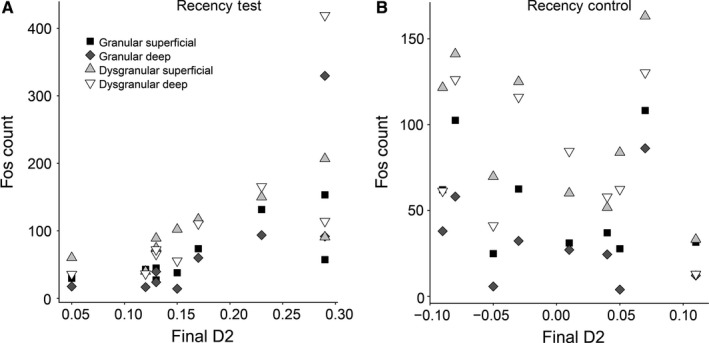
Experiment 2, c‐fos expression associated with recency memory. Scatter plots of the Fos‐positive cell counts and the individual D2 recency memory scores for each of the nine animals in the (A) Recency Test group and (B) Recency Control group. Abbreviations: DysG.Deep: Dysgranular cortex, deep layers; DysG.Sup: Dysgranular cortex, superficial layers; G.Deep, Granular cortex, deep layers; G.Sup: Granular cortex, superficial layers.

The data violated the normality assumption for parametric correlations. Therefore, the relationship between retrosplenial Fos cell counts and behaviour was assessed by Spearman's rank correlation (Table 1). In the experimental group, Fos cell counts in all retrosplenial subarea groupings were significantly positively correlated with D2 (Fig. 9A). This was not the case in the Recency Control group (Fig. 9B). Importantly, Fos cell counts did not correlate with total exploration time in the Recency Test group (Table 1). The Bonferroni corrected alpha value was α = 0.0125.

**Table 1:** Spearman's Rank correlation coefficients comparing recency discrimination (final D2) scores and total exploration time with Fos‐positive cell counts in retrosplenial subareas

	Final D2 score	Total exploration
Recency test		
Granular superficial	*r*_s_ (9) = 0.83, *P* = 0.005^a^	*r*_s_ (9) = 0.03, *P* = 0.93
Granular deep	*r*_s_ (9) = 0.80, *P* = 0.010	*r*_s_ (9) = −0.03, *P* = 0.93
Dysgranular superficial	*r*_s_ (9) = 0.87, *P* = 0.002^a^	*r*_s_ (9) = −0.37, *P* = 0.33
Dysgranular deep	*r*_s_ (9) = 0.92, *P* = 0.001^a^	*r*_s_ (9) = −0.17, *P* = 0.67
Recency control		
Granular superficial	*r*_s_ (9) = −0.08, *P* = 0.83	*r*_s_ (9) = 0.32, *P* = 0.41
Granular deep	*r*_s_ (9) = −0.28, *P* = 0.46	*r*_s_ (9) = −0.57, *P* = 0.11
Dysgranular superficial	*r*_s_ (9) = −0.33, *P* = 0.38	*r*_s_ (9) = −0.62, *P* = 0.08
Dysgranular deep	*r*_s_ (9) = −0.12, *P* = 0.77	*r*_s_ (9) = −0.42, *P* = 0.27

The Bonferroni corrected alpha value is α = 0.0125.

a Denotes significant (*P *<* *0.0125).

##### Inter‐region correlations

Finally, the relationships between Fos‐positive cell counts in the retrosplenial cortex and other regions were examined. Here, we focused on those regions either closely connected with the retrosplenial cortex or implicated in recency memory: the anterior thalamic nuclei, the hippocampus (subiculum) and prelimbic cortex (Fig. 3B). Inspection of the results showed that the corresponding Fos counts in the dorsal subiculum were very low, creating a floor effect. Therefore, only counts for ventral subiculum were analysed.

After correcting for multiple comparisons (α = 0.0125), Fos cell counts in the ventral subiculum in the Recency Test group correlated with those in two of the retrosplenial subregion groupings: Granular deep and Dysgranular deep (Table 2). Consistent with this finding, the Fos cell counts in the ventral subiculum of the Recency Test group also correlated with the D2 scores [*r*_s_ (9) = 0.82, *P = *0.007]. These same subicular Fos counts did not correlate with overall levels of object exploration [*r*_s_ (9) = 0.22, *P = *0.58]. In addition, Fos‐positive cell counts in the prelimbic cortex were significantly correlated with those in Granular Deep retrosplenial cortex.

**Table 2:** Spearman's Rank correlation coefficients for the inter‐regional correlations

	ATN	Ventral subiculum	Prelimbic cortex
Recency test			
Granular superficial	*r*_s_ (9) = 0.55, *P* = 0.13	*r*_s_ (9) = 0.77, *P* = 0.02	*r*_s_ (9) = 0.73, *P* = 0.03
Granular deep	*r*_s_ (9) = 0.62, *P* = 0.08	*r*_s_ (9) = 0.87, *P* = 0.002^a^	*r*_s_ (9) = 0.83, *P* = 0.005^a^
Dysgranular superficial	*r* (9) = 0.32, *P* = 0.41	*r*_s_ (9) = 0.55, *P* = 0.13	*r*_s_ (9) = 0.53, *P* = 0.14
Dysgranular deep	*r*_s_ (9) = 0.55, *P* = 0.13	*r*_s_ (9) = 0.85, *P* = 0.004^a^	*r*_s_ (9) = 0.63, *P* = 0.07
Recency control			
Granular superficial	*r*_s_ (9) = 0.45, *P* = 0.22	*r*_s_ (9) = 0.90, *P* = 0.001^a^	*r*_s_ (9) = 0.62, *P* = 0.08
Granular deep	*r*_s_ (9) = 0.60, *P* = 0.08	*r*_s_ (9) = 0.92, *P* = 0.001^a^	*r*_s_ (9) = 0.70, *P* = 0.04
Dysgranular superficial	*r*_s_ (9) = 0.40, *P* = 0.27	*r*_s_ (9) = 0.60, *P* = 0.09	*r*_s_ (9) = 0.42, *P* = 0.27
Dysgranular deep	*r*_s_ (9) = 0.35, *P* = 0.36	*r*_s_ (9) = 0.60, *P* = 0.09	*r*_s_ (9) = 0.43, *P* = 0.24

ATN, anterior thalamic nuclei.

The Bonferroni corrected alpha value is 0.0125.

a Denotes significant (*P *<* *0.0125).

For the Recency Control group, there were again significant correlations between Fos counts in the retrosplenial cortex (Granular deep and Granular superficial) and the ventral subiculum, and between the retrosplenial cortex (Granular deep) and prelimbic cortex (Table 2).

## General discussion

The present experiments signal the involvement of the rodent retrosplenial cortex in nonspatial recency memory. The recency behavioural tasks are assumed to tax ‘what/when’ information as they assess functions different from those required for object recognition memory, despite the logical possibility that both could be solved by trace strength comparisons (Fortin *et al*., 2002; Barker *et al*., 2007; DeVito & Eichenbaum, 2011; Albasser *et al*., 2012; Cross *et al*., 2012). This distinction is supported by the lesion deficit on the Within‐Block recency task (Experiment 1b), which contrasted with the normal levels of novelty recognition during the two Sample Phases in Experiment 1a, as well as the intact reduction in exploration times between the first presentation of an object in the Sample Phase, when novel, and later during the Test Phase, when familiar (Experiment 1b). Likewise, previous studies have shown that retrosplenial cortex lesions spare the recognition of novel objects (Ennaceur *et al*., 1997; Vann & Aggleton, 2002; see also Bowers *et al*., 1988).

It is notable that similar dissociations between recency (impaired) and recognition memory (spared) are seen in rats following lesions in the anterior thalamic nuclei, the mammillothalamic tract and the medial prefrontal cortex (Hannesson *et al*., 2004b; Wolff *et al*., 2006; Barker *et al*., 2007; Albasser *et al*., 2012; Cross *et al*., 2012; Dumont & Aggleton, 2013; Nelson & Vann, 2016). The same dissociation is also often seen after hippocampal lesions, i.e. impaired recency but spared recognition memory (Cho & Sharp, 2001; Vann & Aggleton, 2004; Pothuizen *et al*., 2008; Harland *et al*., 2014; Nelson *et al*., 2015; Albasser *et al*., 2012). This shared pattern of deficits is striking given the many interconnections between these same sites. In the case of retrosplenial cortex, it has dense connections with both the anterior thalamic nuclei and the hippocampal formation (Wyss & Van Groen, 1992; van Groen *et al*., 1993), though its links with prelimbic cortex are more indirect, e.g. via the anterior cingulate cortex (Hoover & Vertes, 2007) and via the anterior thalamic nuclei (Shibata & Naito, 2005; Hoover & Vertes, 2007).

While rats with retrosplenial cortex lesions were impaired on the Within‐Block recency condition (Experiment 1b), the same rats solved the Between‐Block recency problems (Experiment 1a). These different outcomes were not simply due to changes in task difficulty as the D2 scores of the control group were comparable for both test conditions. As, however, there was no lesion by type of recency test interaction, it is premature to assume that retrosplenial lesions cause rats to be particularly sensitive to distinguishing elements from a continuous series, as is required for Within‐Block recency tests (Templer & Hampton, 2013). A somewhat different perspective is that the retrosplenial lesion deficit only clearly emerged for Within‐Block recency as Between‐Block recency problems can be solved by a more distributed, interconnected network of structures (including retrosplenial cortex) that can better compensate for the loss of one site.

To explore this possibility, a different approach was adopted. Experiment 2 looked at the expression of c*‐fos* in retrosplenial cortex following performance of a Between‐Block recency task. While the absolute levels of retrosplenial Fos did not distinguish the Recency Test group from the Recency Controls, the two groups showed different sets of activity relationships. In the Recency Test group, the numbers of Fos‐positive cells in both the deep and superficial granular and dysgranular retrosplenial cortex were positively correlated with recency discrimination performance. Similar performance‐related Fos correlations were not found in the Recency Control group nor were they found for overall levels of object exploration in the Test Phase, supporting the notion that the retrosplenial activity counts in the experimental group reflected processing that contributed to recency discrimination. More detailed inspection of the results showed that seven of the ten original retrosplenial regions of interest had significant c‐*fos* correlations with D2 recency performance (all *P *<* *0.025, data not presented). Consequently, it was not possible to detect a particular retrosplenial subregion or particular layer (deep or superficial) that had an especially close association with recency performance.

With these correlations in mind, it is helpful to return to the histological results from Experiment 1. In order to minimise the risk of hippocampal damage, the surgeries sometimes spared parts of granular retrosplenial cortex, though all cases involved appreciable dysgranular cell loss. Nevertheless, when the lesion group was divided in two, based on lesion extent, no statistical differences emerged. The results, therefore, suggest that the dysgranular and granular subregions work closely together, such that damage to the former is sufficient to induce performance deficits (see Hindley *et al*., 2014). It should be added that this pattern of cell loss was not related to the apparent sparing of Between‐Block recency. The implication is that the Within‐Block task constrains the ways the task can be solved, leaving it more vulnerable to retrosplenial cortex damage.

The next issue concerns the possible relationships between c*‐fos* activity in retrosplenial cortex and that in other brain sites after between‐block recency testing. This analysis focussed on the hippocampus (the subiculum), the anterior thalamic nuclei and the prelimbic cortex, given their involvement in recency memory (Hannesson *et al*., 2004b; Barker *et al*., 2007; Albasser *et al*., 2012; Dumont & Aggleton, 2013). Of these other sites, only the Fos cell counts in the ventral subiculum correlated with the D2 recency scores which, like the retrosplenial cortex, were not associated with total exploration. In addition, the Fos counts in the ventral subiculum showed significant positive correlations with multiple retrosplenial sites. These correlations were especially consistent with deep retrosplenial layers. It was also found that the Fos cell counts in prelimbic cortex correlated with those from the deep (layers V‐VI) granular retrosplenial cortex. The functional specificity of these retrosplenial relationships requires further examination as there were also retrosplenial – prelimbic cortex and retrosplenial – subiculum correlations in the Recency Control group. The pattern of results suggests that intrinsic relationships between these multiple sites may be sharpened by performing recency discriminations. At the same time, the interconnections that retrosplenial cortex has with both the prelimbic cortex and the ventral subiculum often include indirect steps (Shibata & Naito, 2008; Prasad & Chudasama, 2013), pointing to the involvement of additional sites.

Together, the two experiments support a role for the retrosplenial cortex in recency memory and suggest a link with its hippocampal, thalamic and frontal connections. In addition, the finding that activity in both the superficial and deep cell layers of the retrosplenial cortex was correlated with recency performance implies that relevant interactions could occur with multiple distal sites. For example, cells in the deep retrosplenial layers not only project to the subiculum and postsubiculum in the hippocampal formation (from layer V) but also to the anterior thalamic nuclei (from layer VI) (van Groen & Wyss, 1990, 1992a; Van Groen & Wyss, 2003). Meanwhile, cells in both the deep and superficial layers of the retrosplenial cortex project to cortical association areas (Sripanidkulchai & Wyss, 1987; van Groen & Wyss, 1990, 1992a; Van Groen & Wyss, 2003). The relationship with the ventral subiculum is informative as this region not only has strong projections to prelimbic cortex but the ventral hippocampus also receives many inputs from the perirhinal and lateral entorhinal cortices (Agster & Burwell, 2013), connections presumed to provide object information (Ritchey *et al*., 2015).

As already noted, the present study did not find activity differences between the deep or superficial retrosplenial layers with respect to performance. Meanwhile, previous immediate‐early gene studies of spatial memory have found retrosplenial changes across days of training (Maviel *et al*., 2004; Malinowska *et al*., 2016), including switches in c‐*fos* expression from deep (recent memory) to more superficial (remote memory) layers (Frankland & Bontempi, 2005). In addition, electrophysiological analyses of retrosplenial cortex during the development of avoidance learning have highlighted lamina activity differences with respect to different stages of learning (Gabriel *et al*., 1980, 1991; Gabriel & Talk, 2001). These studies all point to the value of future investigations of nonspatial learning that consider different stages and different time points of learning and retrieval (Katche *et al*., 2013).

The involvement of the retrosplenial cortex in both recency memory and spatial learning leads to the prediction that this area might be of particular importance when these demands intersect. An example would be for those spatial tasks, such as T‐maze alternation, in which the rodent must distinguish one trial from the next, i.e. tests of spatial ‘working memory’ (Olton & Paras, 1979). It, therefore, seems surprising that there is little evidence to support this view. Lesions of retrosplenial cortex typically produce mild deficits on tests of both working and reference spatial memory (Aggleton *et al*., 1995; Whishaw *et al*., 2001; Pothuizen *et al*., 2008; Nelson *et al*., 2015) even though only the former tests particularly tax temporal discriminations. The implication is that temporal signalling for spatial and nonspatial tasks involves different mechanisms. This conclusion is supported by evidence for the opposite dissociation, i.e. impaired spatial but intact object recency, which is found after dorsal subiculum lesions (Potvin *et al*., 2010). In practice, deficits on spatial tasks following retrosplenial lesions are more evident when the rat is required to change its strategy, e.g. from using local to distal cues (Vann & Aggleton, 2004; Nelson *et al*., 2015). Such deficits suggest a more attentional, top‐down role that, like recency memory, echoes prefrontal cortex function. Indeed, a part of the rationale for the present study was to explore possible parallels between retrosplenial cortex and frontal cortex function as lesions in both areas disrupt tests based on the Stroop Task (Haddon & Killcross, 2006; Nelson *et al*., 2014). Furthermore, a potential test of cognitive control, which involves switching sensory modalities to solve recognition problems, is also impaired by retrosplenial cortex lesions (Hindley *et al*., 2014).

The present pattern of results reveal clear similarities with the analysis of a man with left retrosplenial cortex damage, which implied that this area is involved in both Between‐Block and Within‐Block verbal recency judgements but not required for recognition (Bowers *et al*., 1988). To this pattern of temporal deficits can be added to the failure to use topographic information (Epstein & Vass, 2014), which is most consistently associated with right retrosplenial cortex damage in humans (Maguire, 2001; Vann *et al*., 2009). Recent functional imaging studies (Hsieh & Ranganath, 2015) add further weight to the idea that the retrosplenial cortex assists with temporal order information, with activation patterns in both hemispheres that are set within a larger network, including medial prefrontal cortex.

Experiments 1 and 2 provide new evidence that the rat retrosplenial cortex is part of a network of sites normally involved in learning or retrieving temporal order relationships. The finding that the rodent retrosplenial cortex contributes to what/when memory can be added to the many previous studies showing the importance of the same area for spatial memory. The implication is that retrosplenial cortex, by extension, is also important for what/where/when memory, i.e. episodic‐like memory. This interpretation is consistent with the reports of retrosplenial amnesia in humans, which may be strongly linked to a loss of contextual information (Valenstein *et al*., 1987; Maguire, 2001; Vann *et al*., 2009; Miller *et al*., 2014).

## Author contributions

Conceived and designed experiments 1 and 2: ALP, ADJ, LK, CMO, SDV, JPA. Performed experiment 1: ALP, MD. Performed experiment 2: EA, LK, CMO. Analysed the data: ALP. Wrote the paper: ALP, AJD, SDV, JPA.

## Data accessibility

The raw data files for Experiment 1 (RecencyRSC.csv) and Experiment 2 (RecencyFos.csv) will be uploaded with this manuscript on submission.
