# Supplementary material for: The retrosplenial cortex and object recency memory in the rat
Source: Eur J Neurosci. 2017 May 4;45(11):1451–64. doi: 10.1111/ejn.13577 (PMC5488228; doi:10.1111/ejn.13577)
Supplement: Supplementary file 1 [file EJN-45-1451-s001.pdf]

EJN European Journal of Neuroscience FENS Federation of European Neuroscience Societies

The retrosplenial cortex and object recency memory in the rat

Anna L. Powell, Seralynne D. Vann, Cristian M. Olarte-Sánchez, Lisa Kinnavane, Moira Davies, Eman Amin, John P. Aggleton, and Andrew J.D. Nelson

Review timeline:

Submission date:	16 December 2016
Editorial Decision:	30 January 2017
Revision received:	15 February 2017
Editorial Decision:	09 March 2017
Revision received:	24 March 2017
Accepted:	06 April 2017

Editor: Thomas Klausberger

1st Editorial Decision 30 January 2017

Dear Dr. Powell,

Your manuscript was reviewed by external reviewers as well as by the Section Editor, Dr. Thomas Klausberger, and ourselves. The reviews collectively indicate that your experiments generated new and potentially important information. However, there are several issues that need to be addressed before we can consider your manuscript further for publication in EJN. In particular the aim and rationale of the study should be introduced better and several concerns regarding the presentation of the results have been raised by the referees. We assume that these points can be fully addressed in a revised manuscript.

In Addition, please attend to the following issues:
- Please replace bargraphs with scatterplots as per our recent editorial - http://onlinelibrary.wiley.com/doi/10.1111/ejn.13400/full
- The methods & results should not be grouped together and divided by experiment. This needs reformatting.
- As with the Introduction section, it is important to reframe the abstract to better motivate the study

If you are able to respond fully to the points raised, we would be pleased to receive a revision of your paper within 30 days.

Thank you for submitting your work to EJN.

Kind regards,

John Foxe & Paul Bolam
co-Editors in Chief, EJN

Reviews:

Reviewer: 1 (Giuseppe Iaria, University of Calgary)

Comments to the Author

In this manuscript, the Authors aimed at investigating the role of the retrosplenial cortex on the object recency memory in rats. The Authors performed a lesion study followed by a c-fos expression study, and provide complementary evidence supporting the critical role of the retrosplenial cortex in recency memory. I found this study interesting, potentially providing novel findings that may be helpful for a better understanding of the functional properties of the retrosplenial cortex. However, the study presents with a few important issues that the Authors will need to address before the manuscript can be considered for publication in the European Journal of Neuroscience.

First, the Authors should introduce the topic more extensively, and describe the rationale for this study highlighting their specific hypotheses. In this current version, the Introduction is mostly dedicated to the presentation of the study. This part should be removed or moved to the methods section.

Second, the Authors focus their argument of a significant role of the retrosplenial cortex on recency memory based on a single case study. There are however, a significant number of studies supporting the role of the retrosplenial cortex on spatial cognition This should not be ignored.

Third, and this is a major issue to be addressed, it is not clear to me why the Authors decided to adopt two different tasks to evaluate performance in between and within blocks. In one block rats are presented with a pair of old and novel objects; in another block, rats are presented with two identical objects. These two different learning approaches might have contributed significantly to the difference in performance. I understand that the Authors followed the procedure of a previous study, but perhaps it would have been more appropriate to use the same learning approaches to avoid differences in difficulty between tasks. Also, it is not clear to me why the Authors did not perform a single experiment in which only one sample phase would have been used before performing the testing phase; that single sample phase could have been interrupted with a delay, and rats could have been tested for within and between effects. It would be important to replicate the effects by performing this experiment.

Fourth, in the discussion, the Authors should articulate more on the cortical layers and their relevance towards encoding and retrieval phases of the task. In addition, they should link their findings and discuss the relevance of them in the context of previous work done on the topic (and cited in the discussion). Also, the discussion should address more general issues. For instance, is there anything we can learn from this study with respect to a potential left and right dissociation of the retrosplenial cortex in humans?

Reviewer: 2 (Andrew Alexander, Boston University)

Comments to the Author
Powell et al. examined the role of retrosplenial cortex (RSC) in object recency memory (ORM), wherein animals are tested on their ability to distinguish items based on the temporal order of exposure to them. In the first experiment, the authors utilized extensive NMDA lesions of the RSC (both granular and dysgranular) to assess discrimination performance (D2 measure) on the ORM task. RSC lesioned animals were impaired at distinguishing the temporal order of items presented in a continuous series (within-block). but could distinguish between objects that had previously been presented in two discrete blocks (between-block). Exploration time of familiar versus novel objects (in experiment 1b first exposure versus later exposures) in both experiments revealed intact recognition memory for RSC lesioned animals.
In a second experiment, Powell et al. examined c-fos expression in RSC and associated structures as a function of ORM task performance. The authors utilized two experimental groups. The ‘recency test’ group completed discriminations between objects initially sampled from two distinct blocks. In contrast, the ‘recency control’ group discriminated from object pairs that were taken from successive trials within a single sampling block, thus decreasing recency information and making discrimination more difficult. Accordingly, the authors observed that rats in the recency control group were significantly worse at discriminating between object pairs. For animals in the recency test group, RSC and ventral subiculum (vSub) c-fos counts

were positively correlated with D2 scores, suggesting that neuronal activation was correlated with recency task performance. For both test and control groups, vSub c-fos counts were correlated with counts taken from deep layers of RSC (both granular and dysgranular) and prelimbic fos counts were correlated with deep layers of granular RSC.
This work convincingly implicates RSC (and interconnected regions) in recency memory. This is important data, as RSC has a known role in spatial memory, but little is known as to how RSC functions in non-spatial memories despite the fact that human RSC damage results in disruptions of sequential memory. As such, I recommend it for publication given that the following issues are addressed by the authors.

1) It would be helpful if the authors provided more information in the introduction about the logic behind the two different forms of ORM tests. If an RSC lesioned animal cannot perform one or the other, what does that specifically mean about the role of RSC in recency memory? This point may not be clear to someone outside of this field, and it would be great to have this information prior to interpreting the results.

2) This comment is related to discrimination metrics (D1 and D2). Specifically, what constitutes a 'correct' recency discrimination? Could the authors please explain in the introduction or results why preference for the older item indicates recency? This is especially unclear when considering the definition of D1 and D2 in the current manuscript versus similar studies referenced in this work. For example, in the methods of the current paper, the authors describe D1 and D2 as:

"D1 is the time spent exploring the more recent object subtracted from the time spent exploring the older object…Consequently, a positive [D2] score indicates a preference for the older item"

However, in referenced work from the same group (Albasser et al., 2012):

"Index D1 is the duration of exploration time devoted to the novel object minus the exploration time devoted to the familiar object…. A positive D2 index shows a preference for novel objects"

Is it the case that the direction of discrimination is irrelevant? Clarification is needed.

3) Can the authors please provide examples of the c-Fos immunohistology for the ROIs? This is important given the use of automated cell counting software.

4) In figure 6A, the ROI for caudal dysgranular RSC does not appear to cover the full extent of the region. Was this intentional? If the entirety of dysgranular RSC was included would it influence the interaction between RSC region and c-Fos counts?

5) Figure 9. Although the authors acknowledge it in the figure legend, the x-axes in this figure are misleading. The D2 scores for individual animals are not equally spaced yet the data points are presented as though they are. This gives the reader the impression that the relationship between D2 score and c-Fos count in Figure 9B is more linear than in actuality. The data on the x-axis should not be discretized or the visualization should be changed.

Minor Issues:
- Figure 9A and B aren't labeled on the figure.
- In the results section for Experiment 1b: does 'trial type' refer to the number of interleaving items? If so, could this be explained more explicitly?

EJN European Journal of Neuroscience FENS Federation of European Neuroscience Societies

Authors' Response 15 February 2017

Editor's comments

- The aim and rationale of the study should be introduced better.
  As is explained below, we have added further explanation of both the rationale and the predictions associated with the present study.
- Please replace bargraphs with scatterplots as per our recent editorial
  In accordance with these instructions the relevant figures have been revised.
- The methods & results should not be grouped together and divided by experiment. This needs reformatting.
  The methods and results have now been reformatted accordingly. (One consequence is that the old Figure 6 is now Figure 3, so that the old Figures 3-5 have all be renumbered one higher.)
- As with the Introduction section, it is important to reframe the abstract to better motivate the study.
  Information has been added to the Abstract to strengthen the rationale for the study.

Reviewer: 1

1. Introduce the topic more extensively, and describe the rationale / hypotheses.

We hope that the changes made to the text of the Introduction will make the rationale and hypotheses of this study more evident to the reader. We have added further information about what is known about the functions of the rodent retrosplenial cortex (e.g., see #3) and added more information concerning different classes of temporal order discriminations. In addition, we have added the following paragraph:

"Given the dense interconnectivity between the retrosplenial cortex, hippocampus, and anterior thalamic nuclei, alongside the involvement of the latter two regions in recency memory, we anticipated that temporal order discriminations would be affected by retrosplenial cortex lesions (Experiment 1). Moreover, it was predicted that such recency judgements would engage the retrosplenial cortex, as measured by c-*fos* expression, with related changes in interconnected target sites."

2. In this current version, the Introduction is mostly dedicated to the presentation of the study. This part should be removed or moved to the methods section.

In response to the Reviewer's comments we have removed much of the more detailed description of the task design. We have retained a minimal amount of information that helps to clarify the nature of the different behavioural tasks.

The deleted text from the Introduction is shown below (struck out).

~~To mark the end of the first block, the rat was removed from the apparatus, before being returned and presented with the second block of objects. During the subsequent test trials the rat was simultaneously presented with a single object from each block of sample trials.~~

~~The procedure for the second group was designed to match the sensorimotor demands of the recency test ('Recency Control' group). For this control task, the stimuli in the final phase were presented as if for a Within-Block recency test, except that the object pairings were intended to be insoluble, i.e., the rats could not discriminate the objects on the basis of their temporal order.~~

3.There are a significant number of studies supporting the role of the retrosplenial cortex on spatial cognition. This should not be ignored.

The primary aim of the study was, of course, to look at *non-spatial* functions of the retrosplenial cortex. Nevertheless, in paragraph one of the Introduction we have added more information concerning the importance of the retrosplenial cortex for spatial cognition (see below). Likewise, in the Discussion we specifically consider whether the deficits we see in sequence memory carry over to spatial working memory tasks that involve distinguishing order.

Introduction, paragraph 1:
"It has long been appreciated that this interlinked network appears vital for rodent spatial memory. Lesions of the retrosplenial cortex impair tests of spatial memory that require the use of allocentric cues, with examples coming from tasks in the water maze and radial-arm maze, as well as tasks that rely on egocentric directional information and path integration (Sutherland et al., 1988; Warburton et al., 1998; Pothuizen et al., 2008; Keene & Bucci, 2009). Temporary inactivation studies using the radial-arm maze further implicate the retrosplenial cortex in spatial working memory (Cooper & Mizumori, 1999; Cooper et al., 2001), while electrophysiological recordings reveal a role in navigation (Cho & Sharp, 2001). However, the importance of the rodent retrosplenial cortex for nonspatial memory remains uncertain."

4.It is not clear to me why the Authors decided to adopt two different tasks to evaluate performance In-Between and Within blocks. Perhaps it would have been more appropriate to use the same learning approaches to avoid differences in difficulty between tasks.

Additional references (e.g., DeVito & Eichenbaum, 2011) have been provided to support the likelihood of a qualitative distinction between these two ways of solving order discriminations.
Whilst, in some studies the Within-Block task is more difficult to solve than the Between-Block version, this problem did not seem to occur in the present study as the discrimination indices (D2 scores) of the control group were comparable for the two test conditions. This point is explained in the Discussion.

5.Also, it is not clear to me why the Authors did not perform a single experiment in which only one sample phase would have been used before performing the testing phase; that single sample phase could have been interrupted with a delay, and rats could have been tested for within and between effects. It would be important to replicate the effects by performing this experiment.

This proposal appears to provide an elegant solution to matching the two tasks more closely. (Although as already noted, the two tasks in Experiment 1a and 1b had comparable recency index scores, i.e., in this critical sense they were matched).

When trying to design the suggested experiment, a number of practical problems emerge. The first relates to the fact that a key strength of the bow-tie maze procedure is that it provides multiple trials in order to reduce variance. The total number of trials required when combining experiments and including both Sample and Test Phases soon becomes counter-productive. One consequence is that the animals start to explore less and less. Another concerns is that the additional build-up of proactive interference might introduce further factors affecting performance as the test proceeds. A further point is that DeVito & Eichenbaum (2011) reported that mice might begin to treat these two problems very differently, only performing recency discriminations in one condition (Within-Block), highlighting the value of separating these procedures.

A final point is that Experiment 2 had to use just one procedure (otherwise the results would have been ambiguous). For this reason, it was important to separate out the relevant procedures in Experiment 1 in order to provide a match for Experiment 2.

6. Need to articulate more on the cortical layers and their relevance towards encoding and retrieval phases of the task. We have now added the following to the Discussion.

"As already noted, the present study did not find activity differences between the deep or superficial retrosplenial layers with respect to performance. Meanwhile, previous immediate-early gene studies of spatial memory have found retrosplenial changes across days of training (Maviel et al., 2004; Malinowska et al., 2016), including switches in c-fos expression from deep (recent memory) to more superficial (remote memory) layers (Frankland & Bontempi, 2005). In addition, electrophysiological analyses of retrosplenial cortex during the development of avoidance learning have highlighted lamina activity differences with different stages of learning (Gabriel et al., 1980; 1991; Gabriel & Talk, 2001). These studies all point to the value of future investigations of nonspatial learning that consider different stages and different time points of learning and retrieval (Katche et al., 2013)."

7. Link their findings and discuss the relevance of them in the context of previous work done on the topic (and cited in the discussion) (e.g. is there anything we can learn from this study with respect to a potential left and right dissociation of the retrosplenial cortex in humans?)

We have now added more information concerning the context of our results in the Discussion. It is true that in the original manuscript we had said nothing about right/left hemispheric differences in humans, but this reflected our natural caution about drawing such inferences from a study of the rodent brain. That said, there is clearly the possibility that in the human retrosplenial cortex there is a right/left specialisation for space/topography and recency, respectively. This possibility is now noted at the end of the Discussion.

"The present findings reveal similarities with the analysis of a man with left retrosplenial cortex damage, showing that this area is involved in both Between-Block and Within-Block verbal recency judgements (Bowers et al., 1988). This pattern of temporal deficits can be added to the failure to use topographic information (Epstein & Vass, 2015), which is most consistently associated with right retrosplenial cortex damage in humans (Maguire, 2001; Vann et al., 2009). Recent functional imaging studies (Hseih & Ranganath, 2015) add further weight to the idea that the retrosplenial cortex assists with temporal order information, with activation patterns in both hemispheres that are set within a larger network, including medial prefrontal cortex. This pattern of results is consistent with key elements of the present study."

Reviewer 2

1) It would be helpful if the authors provided more information in the introduction about the logic behind the two different forms of ORM tests. If an RSC lesioned animal cannot perform one or the other, what does that specifically mean about the role of RSC in recency memory? This point may not be clear to someone outside of this field, and it would be great to have this information prior to interpreting the results.

We hope that the addition of the following text to the introduction has helped to clarify this point:

"Other studies with intact mice (DeVito & Eichenbaum, 2011) further support the notion that these two types of recency judgement can rely on qualitatively different mnemonic processes. For example, it maybe that objects presented in discrete blocks are additionally grouped by their block, allowing 'Between-Block' recency judgements to be less reliant on detailed sequential information than 'Within-Block' judgements (DeVito & Eichenbaum, 2011; Templer & Hampton, 2013). The implication is that different systems might support these two classes of temporal information. Consequently, rats with retrosplenial cortex lesions completed both classes of recency test."

2) This comment is related to discrimination metrics (D1 and D2). Specifically, what constitutes a 'correct' recency discrimination? Could the authors please explain in the introduction or results why preference for the older item indicates recency? This is especially unclear when considering the definition of D1 and D2 in the current manuscript versus similar studies referenced in this work. For example, in the methods of the current paper, the authors describe D1 and D2 as: "D1 is the time spent exploring the more recent object subtracted from the time spent exploring the older object. Consequently, a positive [D2] score indicates a preference for the older item" However, in referenced work from the same group (Albasser et al., 2012): "Index D1 is the duration of exploration time devoted to the novel object minus the exploration time

devoted to the familiar object. A positive D2 index shows a preference for novel objects" Is it the case that the direction of discrimination is irrelevant? Clarification is needed.

We hope that the highlighted changes made to the text in the Method section clarify this point, in particular, the addition below:

"In standard tests of object recognition, the greater time spent exploring a novel object when compared to a familiar object is used as a proxy measure of successful recognition memory. Here, when testing recency memory, the contrast is made between the exploration times for the older object (i.e. treated as if it were the "novel" object) with those of a more recently presented object (i.e. treated as if it were the "familiar" object). This contrast follows numerous experiments that have confirmed how rats prefer to explore the least familiar object of a pair (Hannesson, Vacca, et al., 2004; Mitchell & Dalrymple-Alford, 2005; DeVito & Eichenbaum, 2011; Albasser et al., 2012)."

3) Can the authors please provide examples of the c-Fos immunohistology for the ROIs? This is important given the use of automated cell counting software.

Please see the updated version of Figure 6 (now Figure 3) where we have added stained sections.

4) In figure 6A, the ROI for caudal dysgranular retrosplenial cortex does not appear to cover the full extent of the region. Was this intentional? If the entirety of dysgranular cortex was included would it influence the interaction between retrosplenial cortex and c-Fos counts?

Apologies, this was a formatting error that has been amended. We did indeed count along the full length of dysgranular cortex. (Note, now Figure 3)

5) Figure 9. Although the authors acknowledge it in the figure legend, the x-axes in this figure are misleading. The D2 scores for individual animals are not equally spaced yet the data points are presented as though they are. This gives the reader the impression that the relationship between D2 score and c-Fos count in Figure 9B is more linear than in actuality. The data on the x-axis should not be discretized or the visualization should be changed.

We have modified the axes on this figure accordingly.

Minor Issues:

• Figure 9A and B aren't labelled on the figure.

This has been corrected.

• In the results section for Experiment 1b: does 'trial type' refer to the number of interleaving items? If so, could this be explained more explicitly?

Yes, it is indeed the case that trial type refers to the number of interleaving object presentations. We have now explicitly stated this in the Results section.

2nd Editorial Decision 09 March 2017

Dear Dr. Powell,

Your revised manuscript was re-evaluated by the two external reviewers as well as by our editorial team. Based on these reviews, we regret to inform you that we are not able to accept your manuscript for publication in EJN in its present form. However, we would be prepared to consider yet another revised

version. For this, we encourage you to implement the changes suggested by the referees, in particular to explain better the rationale of the study and discuss the relevance with respect to the existing literature in a broader way.

Additional queries are indicated in the comments below.

If you are able to respond fully to the points raised, we shall be pleased to receive a revision of your paper within 30 days.

Thank you for submitting your work to EJN.

Kind regards,

John Foxe & Paul Bolam
co-Editors in Chief, EJN

Reviews:

Reviewer: 1 (Giuseppe Iaria, University of Calgary)

Comments to the Author
In this revised version of the manuscript, the Authors have addressed some of my concerns (mostly in the responses to the Reviewers' comments). However, the changes made in the revised manuscript were not significant in terms of framing the study's rationale and results appropriately within the literature. The introduction is still focusing mostly on the Authors' present experiments, and the discussion is not articulating enough on the value of the present findings within the existing literature. In sum, the manuscript has not been significantly changed for the benefit of the readers.

Reviewer: 2 (Andrew Alexander, Boston University)

Comments to the Author
The authors have sufficiently addressed my concerns in their revisions. They have clarified the rationale behind the different ORM tests and discrimination metric. My one remaining issue is that c-Fos examples should be provided for all ROIs, not simply RSC.

Authors' Response 24 March 2017

Reviewer: 1

Comments to the Author
In this revised version of the manuscript, the Authors have addressed some of my concerns (mostly in the responses to the Reviewers' comments). However, the changes made in the revised manuscript were not significant in terms of framing the study's rationale and results appropriately within the literature. The introduction is still focusing mostly on the Authors' present experiments, and the discussion is not articulating enough on the value of the present findings within the existing literature. In sum, the manuscript has not been significantly changed for the benefit of the readers.

The referee wished that the rationale and relevant literature were provided in more depth. To this end, we have both added extra references (8 in total) and have clarified the purpose of the experiment in more detail. In particular, we have explained how the experiments target a specific aspect of memory ("what/when" information). This particular type of information is highly relevant to episodic-like memory in

rodents. The references have added weight to both the anatomical and functional claims in the Introduction and Discussion. Please see underlined changes in the text of the manuscript.

Reviewer: 2

Comments to the Author
The authors have sufficiently addressed my concerns in their revisions. They have clarified the rationale behind the different ORM tests and discrimination metric. My one remaining issue is that c-Fos examples should be provided for all ROIs, not simply RSC.

Referee 2: As requested, we have added extra photomicrographs to show the appearance of the Fos positive cells in the subiculum, prelimbic cortex, and anterior thalamic nuclei (new Figure 3D). The figure legend has been revised accordingly.
